# Efficacy of sublingual and oral vitamin B12 versus intramuscular administration: insights from a systematic review and meta-analysis

**DOI:** 10.3389/fphar.2025.1602976

**Published:** 2025-12-19

**Authors:** Marta Mazur, Artnora Ndokaj, Claudia Salerno, Jessica Vallone, Roman Ardan, Sabina Bietolini, Florence Carrouel, Aleksandra Wilk, Rachel Sarig, Livia Ottolenghi, Denis Bourgeois

**Affiliations:** 1 BeSSA Department – Interdisciplinary Department of Well-being, Health and Environmental Sustainability, Sapienza University of Rome, Rieti, Italy; 2 Department of Interdisciplinary Dentistry, Pomeranian Medical University, Szczecin, Poland; 3 Department of Oral and Maxillofacial Sciences, Sapienza University of Rome, Rome, Italy; 4 Department of Biomedical, Surgical and Dental Sciences, University of Milan, Milan, Italy; 5 Department of Restorative, Preventive and Pediatric Dentistry, School of Dental Medicine, University of Bern, Bern, Switzerland; 6 Department of Economic Sciences, Koszalin University of Technology, Koszalin, Poland; 7 Department of Humanities, Motor Sciences and Education, Niccolò Cusano University (UNICUSANO), Rome, Italy; 8 Health Systemic Process Laboratory (P2S), UR4129, University Claude Bernard Lyon 1, University of Lyon, Lyon, France; 9 Department of Histology and Embryology, Pomeranian Medical University, Szczecin, Poland; 10 Department of Oral Biology, The Goldschleger School of Dental Medicine, Gray Faculty of Medical and Health Sciences, Tel Aviv University, Tel Aviv, Israel

**Keywords:** vitamin B12, efficacy, bioavailability, therapeutic approaches, meta-analysis, cobalamin

## Abstract

**Background:**

Vitamin B12 deficiency is a widespread condition, particularly among elderly individuals, patients with malabsorption syndromes, and those following plant-based diets. This systematic review and meta-analysis aimed to evaluate and compare the effectiveness of sublingual and oral vitamin B12 administration in comparison with intramuscular (IM) injections, both for improving serum cobalamin and reducing homocysteine levels.

**Methods:**

A comprehensive search was conducted across PubMed, Scopus, and Embase up to July 2024. Eligible studies included randomised controlled trials, cohort, and case-control studies assessing B12 supplementation efficacy via oral, sublingual, or IM administration routes. Meta-analyses were performed using random-effects models. Subgroup analyses evaluated four key factors: administration route efficacy, daily dosage, age group and clinical conditions that may affect vitamin B12 metabolism (e.g., underlying pathology). Sixteen studies were included in the quantitative synthesis, comprising a total of 6,098 participants.

**Results:**

Vitamin B12 supplementation was associated with a significant increase in serum cobalamin levels across all routes of administration (pooled mean difference = +402.6 pg/mL; 95% CI: 293.6 to 511.5; p < 0.001). Homocysteine levels were also significantly reduced across all groups (pooled mean difference = −4.83 μmol/L; 95% CI: −6.55 to −3.11; p < 0.001). No statistically significant differences were observed between oral, sublingual, and intramuscular (IM) routes of administration (p = 0.270 for cobalamin levels and p = 0.485 for homocysteine levels), nor between randomised controlled trials and observational studies (p = 0.268 for cobalamin levels). No dose-response effect was observed (p = 0.485), suggesting that absorption efficiency rather than dosage may be the determining factor. Subgroup analyses by age and clinical conditions (e.g., gastrectomy, unspecified deficiency) revealed comparable efficacy across populations. However, heterogeneity was substantial (I^2^ > 80% in most comparisons), and Egger’s test indicated potential publication bias. Given the high heterogeneity, further studies are needed to confirm the results.

**Conclusion:**

Sublingual and oral B12 supplementation appear to be as effective as intramuscular (IM) injections in improving cobalamin status and reducing homocysteine levels. Given their non-invasive nature, accessibility, and cost-effectiveness of sublingual formulations, they may represent a promising approach for long-term B12 management, particularly in patients with impaired absorption or in resource-limited settings. Further high-quality RCTs are warranted to refine dosing strategies and confirm long-term outcomes.

**Systematic Review Registration:**

CRD42024554513.

## 1 Introduction

Vitamin B12, or cobalamin, is a water-soluble vitamin, that plays an essential role in cellular metabolism, particularly in DNA synthesis, methylation processes, and mitochondrial functions (Green et al., 2017). It is synthesized exclusively by micro-organisms (e.g., *Lactobacillus spp*) (LeBlanc et al., 2013), which are also found in the gastrointestinal tract of herbivorous animals, where the synthesis of B12 occurs and the vitamin can be acquired in animal tissues. An omnivorous diet allows for the B12 intake through animal products (with a bioavailability ranging from 24% to 36% in eggs, up to 65% in lean meat) (Doets et al., 2013), while vitamin B12 is scarce or absent in diets including little or no animal products. It acts as a cofactor for methionine synthase and methylmalonyl-CoA mutase, enzymes that are critical for one-carbon metabolism as well as the catabolism of odd-chain fatty acids and branched-chain amino acids (Rucker et al., 2007).

Clinically overt vitamin B12 deficiency, characterized by hematological and neurological dysfunctions, is relatively uncommon. It typically presents as macrocytic megaloblastic anemia and neurological impairments, including sensory and motor disfunctions, predominantly in the lower extremities, ataxia, and in more advanced stages cognitive decline progressing to dementia, as well as psychiatric disturbances (Green et al., 2017). Beyond systemic symptoms, vitamin B12 deficiency is also associated with a range of oral manifestations, including burning mouth syndrome, recurrent aphthous ulcers, mucosal inflammation, and trigeminal nerve-related pain, which are often underdiagnosed or misattributed to other conditions (Bao et al., 2021; da Silva et al., 2022; Sanjay et al., 2022; Boukssim and Chbicheb, 2024; Field et al., 1995).

Subclinical B12 deficiency, however, is far more prevalent, affecting up to 26% of the general population. It is especially common among older adults, individuals with specific health conditions, children, or those following predominantly or exclusively plant-based diets. This condition may arise due to insufficient dietary intake (resulting from several factors, including: a) the exclusive presence of B12 in animal-derived foods; b) the widespread use of antibiotics in animal farming, which impairs B12 production by the animal gut microbiota; and c) irregular dietary patterns), reduced bioavailability (impairment of the intrinsic factor-mediated absorption pathway, often due to the widespread and prolonged use of proton pump inhibitors), and malabsorption syndromes.

The latter may be caused by intestinal disorders, infections, bariatric surgery, or pharmacological interference, including histamine H2 receptor antagonists and metformin. Additionally, chronic conditions such as HIV and tuberculosis have been implicated in B12 deficiency due to increased metabolic demand and altered absorption mechanisms (Green, 2017).

The diagnosis of vitamin B12 deficiency typically relies on low total B12 levels and elevated concentrations of homocysteine (Hcy), as well as levels of transcobalamin-bound B12 (holoTC) and methylmalonic acid (MMA), which serve as functional indicators of cobalamin insufficiency (Carmel and Sarrai, 2006).

Vitamin B12 requirements fluctuate throughout the life course, influenced by metabolic demand and physiological changes. A safe serum range is 200–350 ng/L, depending on age and clinical condition. The European recommended daily intake also varies by age, beginning at 1.5 μg for infants and increasing to 4 μg for adults, with slightly higher requirements during pregnancy and lactation (4.5 μg and 5 μg, respectively; see Table 1) (EFSA NDA Panel (EFSA Panel on Dietetic Products, Nutrition and Allergies), 2015).

**TABLE 1:** Vitamin B12 Adequate Intake [AI] across life course (EFSA NDA Panel (EFSA Panel on Dietetic Products, Nutrition and Allergies), 2015; McGuire, 2010).

Age class	VIT. B12 μg/day-AI - Europeans	Age class	VIT. B12 μg/day-AI - Americans
7–11 months	1.5	7–11 months	0.5
1–3 years	1.5	1–3 years	0.9
4–6 years	1.5	4–8 years	1.2
7–10 years	2.5	9–13 years	1.8
11–14 years	3.5	14–18 years	2.4
15–17 years	4	≥18 years	2.4
≥18 years	4	Pregnancy	2.6
Pregnancy	4.5	Lactation	2.8
Lactation	5		

Comparable age groupings are proposed in the Dietary Guidelines for Americans, though the recommended intake levels differ, beginning at 0.5 μg in infancy, increasing to 2.4 μg for adults, and rising modestly during pregnancy and lactation (2.6 μg and 2.8 μg, respectively; see Table 1) (McGuire, 2010).

While European guidelines recommend up to 4 μg/day for adults, most of the included studies administered pharmacological doses ranging from hundreds to thousands of micrograms. This significant discrepancy between physiological requirements and therapeutic doses highlights the need for a deeper understanding of B12 absorption dynamics, rather than focusing solely on intake quantity.

Vitamin B12 deficiency is increasingly recognised as a global public health concern, particularly in developing countries and among high-risk populations. A WHO consultation identified the most vulnerable groups as elderly individuals, infants, preschool-aged children, and pregnant or lactating women, due to either increased nutritional demands or impaired absorption capacity (de Benoist, 2008).

Addressing the prevention of B12 deficiency and its public health implications requires a comprehensive, life-course approach based on integrated strategies targeting early detection, dietary optimisation, and effective supplementation, in order to mitigate the long-term consequences of inadequate cobalamin status (Organization, 2000). From a health economics perspective, lifelong B12 supplementation should involve cost-effective delivery routes and tailored dosages to optimise clinical outcomes while minimising excess. Although rare, adverse effects of high-dose vitamin B12 have been reported, including allergic reactions such as fever, rash, itching, hot flushes, dizziness, and nausea, across all cobalamin forms and administration routes (Caballero et al., 2007).

Intramuscular (IM) injection is the most invasive and costly administration method, requiring trained personnel and clinical visits. Oral supplementation is more affordable and accessible but may be ineffective in individuals with gastric malabsorption. Sublingual B12, which bypasses the gastrointestinal tract, is the least invasive and potentially most cost-effective option, especially for individuals with impaired absorption, making it an attractive alternative for long-term and patient-friendly management (Houle et al., 2014; Vidal-Alaball et al., 2006).

Supplementation may involve various forms of cobalamin: adenosylcobalamin, cyanocobalamin, methylcobalamin, or hydroxocobalamin. Cyanocobalamin is the most widely used form due to its chemical stability, although it is not active in its original form. In humans, cyanocobalamin must undergo intracellular enzymatic conversion to the active coenzyme forms—methylcobalamin and adenosylcobalamin—which, depending on the pH environment, can interconvert with hydroxocobalamin (Gherasim et al., 2013), the most common B12 form in both food and human plasma (Obeid et al., 2015).

Despite extensive biochemical knowledge of cobalamin metabolism and its physiological importance, the optimal supplementation route for maintaining or restoring adequate vitamin B12 levels remains debated. Previous studies have reported inconsistent results when comparing oral, sublingual, and intramuscular administration, often limited by small sample sizes or heterogeneous populations (Houle et al., 2014; Vidal-Alaball et al., 2006; Gherasim et al., 2013; Obeid et al., 2015). Therefore, a comprehensive synthesis of available evidence is needed to clarify whether non-invasive routes provide efficacy comparable to intramuscular therapy.

The aim of this systematic review and meta-analysis was to critically assess and compare the effectiveness of different vitamin B12 administration routes in correcting deficiency.

## 2 Materials and methods

### 2.1 Protocol and registration

This systematic review was conducted in accordance with the Preferred Reporting Items for Systematic Reviews and Meta-Analyses (PRISMA) statement (Moher et al., 2009) and the guidelines from the Cochrane Handbook for Systematic Reviews of Interventions (Higgins and Green, 2008). PRISMA checklist is provided in Supplementary Table S1. The systematic review protocol was registered in the International Prospective Register of Systematic Reviews (PROSPERO), registration number: CRD42024554513 (Available from) (NIHR, 2025).

### 2.2 PICOs question

The primary research question addressed in this review was: “What is the effectiveness of sublingual/oral administration of Vitamin B12 alone or compared to other forms of administration (e.g., intramuscular injection)?”. The PICOs elements were defined as follows:

P (Participants): subjects of any age diagnosed with Vitamin B12 deficiency.
I (Intervention): sublingual/oral administration of Vitamin B12.
C (Comparison): intramuscular administration of Vitamin B12.
O (Outcome):
  ‐ Primary outcome: changes in serum Vitamin B12 levels, clinical improvement of Vitamin B12 deficiency symptoms.
S (Study design): Randomised controlled trials (RCTs), non-randomised controlled trials (N-RCTs), cohort studies, and case-control studies.

### 2.3 Information sources and search strategy

Three databases (PubMed, Embase, and Scopus) were searched up to 15 July 2024.

The search strategy was initially developed for PubMed using keywords and MeSH terms and was then adapted to the other databases. Search strings used for each database are presented in Supplementary Table S2. Reference lists of included studies and relevant reviews were screened manually to identify additional eligible studies. Rayyan software (Ouzzani et al., 2016) was employed for reference management, including the identification and removal of duplicate entries.

### 2.4 Study selection, eligibility criteria, and data extraction

Reviewers underwent training at each step of the process, and a pilot screening was conducted to ensure consistency in applying the eligibility criteria. Three authors (MM, AN, and JV) independently screened titles and abstracts following de-duplication. Disagreements were resolved through discussion or by consultation with a fourth author (CS).

The same three authors proceeded to full-text screening, with any discrepancies resolved in the same manner.

The inclusion and exclusion criteria were defined in direct accordance with the PICOS framework. Specifically, eligible studies included participants (P) with a confirmed diagnosis of vitamin B12 deficiency, interventions (I) involving oral or sublingual vitamin B12 administration, comparators (C) using intramuscular supplementation, and outcomes (O) reporting changes in serum vitamin B12 or homocysteine levels. Only clinical study designs (S)—randomised controlled trials (RCTs), non-RCTs, cohort, or case-control studies—were included.

Inclusion criteria were: (a) RCTs; (b) N-RCTs; (c) case-control studies; (d) cohort studies; (e) cross-sectional studies with no time restriction, (f) only studies published in English were included due to resource limitations and to ensure accuracy in data extraction and risk-of-bias assessment.

Exclusion criteria were: (a) *in-vitro* RCTs; (b) lack of effective statistical analysis; (c) abstract and author debates or editorials; (d) review articles or non-clinical studies; (e) case reports and case series, ongoing and unpublished studies; (g) studies lacking a comparator group.

This review included both RCTs and observational studies (N-RCTs, cohort, and case-control) to assess the efficacy of various Vitamin B12 administration routes. While RCTs remain the gold standard for intervention assessment, observational studies provide complementary evidence, especially when using objective biomarkers such as serum Vitamin B12, MMA, and homocysteine. This approach is consistent with established methodologies adopted in previous systematic reviews assessing micronutrient interventions.

For each eligible study, two authors (MM and AN) independently extracted data, which was then reviewed by a third author (JV) using a piloted spreadsheet, in accordance with Cochrane Collaboration guidelines (Higgins and Green, 2008).

Where relevant data were missing, attempts were made to contact corresponding authors. Studies for which no response was received were excluded.

The following data were collected: publication year, country and continent, study setting (e.g., hospital, private practice, academic clinic), sample size, age range, mean age, study design, population characteristics, intervention details, outcomes, and effect measures (e.g., serum B12 levels). Subgroup analyses were planned based on age group (e.g., children, adults, older adults), baseline serum Vitamin B12, and the form of Vitamin B12 used.

### 2.5 Quality assessment and risk of bias

In line with PRISMA guidelines, the assessment of methodological quality was conducted to evaluate the strength of evidence, recognising that methodological limitations may introduce bias.

For randomised clinical trials, the Jadad scale (Jadad et al., 1996), as used to assign a quality score ranging from 0 to 5, based on randomisation, blinding, and withdrawal reporting. A score of ≥3 was considered indicative of good quality.

To complement the Jadad score, the Cochrane Risk of Bias Tool (RoB 2) (Sterne et al., 2019) was also used for RCTs to provide a domain-based qualitative evaluation, assessing risks related to selection, performance, detection, attrition, and reporting.

For observational studies (cohort and case-control), the Newcastle–Ottawa Scale (NOS) (La Torre et al., 2014), was applied. This tool evaluates selection, comparability, and exposure or outcome assessment. A maximum of nine stars could be awarded, with a higher number indicating better quality.

Risk of bias was interpreted as follows: i) Low risk: All criteria met or no more than one rated as unclear; ii) Moderate risk: Two criteria rated as unclear, or one criterion not met; iii) High risk: Three or more criteria rated as unclear or at least two criteria not met.

The Grading of Recommendation, Assessment, Development, and Evaluation (GRADE) tool was used to assess the certainty of the evidence for the two primary biochemical outcomes—serum vitamin B12 concentration increase and homocysteine reduction—across all included studies, and separately for randomised controlled trials (RCTs) and observational studies.

### 2.6 Meta-analysis

Meta-analysis was performed using the R statistical program, R version 4.4.2 (The R Foundation for Statistical Computing, Wirtschaftsuniversitä̈t Wien, Vienna, Austria) (Sterne et al., 2016; Viechtbauer, 2010), employing a random-effect model via the metafor R package (Del, 2015). The mean change was used as effect estimate. Heterogeneity was assessed quantitatively using the I2-statistics and Cochran’s Q (Higgins and Thompson, 2002). Results were considered statistically significant at p < 0.05. Publication bias was evaluated using a funnel plot and Egger’s test for asymmetry (Egger et al., 1997).

## 3 Results

### 3.1 Study selection and characteristics

**FIGURE 1:**
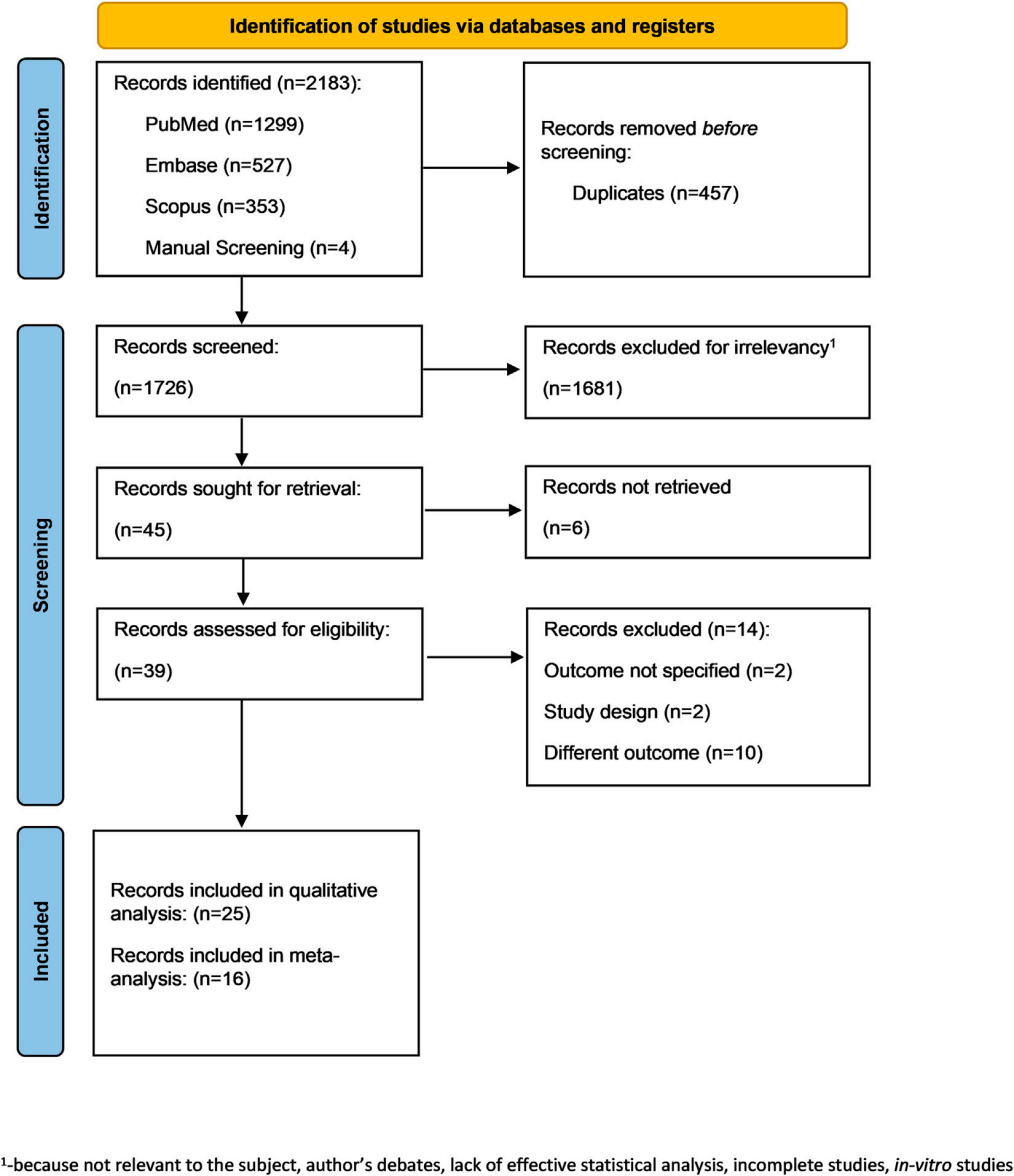
The flow diagram of the search.

The search strategy identified 2,183 potential articles: 1,299 from PubMed, 353 from Scopus, 527 from Embase, 4 from manual screening. After removal of duplicates, 1762 articles were screened. Subsequently, 1717 articles were excluded because they did not meet the inclusion criteria. Of the remaining 45 articles, 6 full texts could not be retrieved and 14 were excluded as they were out of scope. The remaining 25 articles were included in the qualitative synthesis, and 16 in the quantitative synthesis (Figure 1).

The studies included in the qualitative synthesis (Table 2) (Tanaka et al., 1981; Yamane et al., 1995; Kuzminski et al., 1998; Altay et al., 1999; Adachi et al., 2000; Bolaman et al., 2003; Sharabi et al., 2003; Roth and Orija, 2004; Yazaki et al., 2006; Castelli et al., 2011; Kim et al., 2011; Tillemans et al., 2014; Saraswathy et al., 2012; Parry-Strong et al., 2016; Metaxas et al., 2017; Schijns et al., 2018; Sezer et al., 2018; Bensky et al., 2019; Arıcan et al., 2020; Sanz-Cuesta et al., 2020; Tuğba-Kartal and Çağla-Mutlu, 2020; Orhan Kiliç et al., 2021; Ramos et al., 2021; Tandon et al., 2022; Korpeti et al., 2023) were published between 1981 and 2024 and comprised 13 randomised controlled trials, 2 case control study and 10 cohort studies (6 retrospective and 4 prospective). A total of 6,098 participants were included (mean: 244).

**TABLE 2:** Characteristics of the included studies.

Nr	Year	Author	Type of study	Year of the trial	Country	Continent	Study setting	Health status	Mean age (years)	SD age	Age range (years)	Total sample	Male	Female	B12 formulation
1	1981	Tanaka	RCT	1981	Japan	Asia	University hospital	Healthy men			24–44	22	22		Cyanocobalamin, methylcobalamin, adenosylcobalamin, hydroxocobalamin
2	1995	Yamane	Case-control	1990–1992	Japan	Asia	Hospital	Chronic multiple peripheral neuropathy			18–65	44	25	19	Methylcobalamin
3	1998	Kuzminski	RCT	1993–1996	United States	North America	Hospital	VB12 deficiency	71	11		33	7	25	Cyanocobalamin
4	1999	Altay	Retrospective		Turkey	Asia	Hospital	Megaloblastic anemia	11.7	6.4		12			Cyanocobalamin
5	2000	Adachi	Case-control		Japan	Asia	University hospital	Gastrectomy	57.2			31	23	8	
6	2003	Bolaman	RCT	1999–2003	Turkey	Asia	University hospital	Megaloblastic anemia	>16			42			Cyanocobalamin
7	2003	Sharabi	RCT		Israel	Asia	University hospital	VB12 deficiency				30			Cobalamin
8	2004	Roth	Retrospective		United States	North America	Hospital		69	12		60			
9	2006	Yazaki	RCT		United States of America	North America	University hospital	VB12 deficiency	50–80		50–80	41			Methylcobalamin
10	2011	Castelli	RCT	2009–2010	United States	North America	Hospital	VB12 deficiency	52.2	15.3		50	11	39	Cyanocobalamin
11	2011	Kim	Prospective	2008/2011	Corea	Asia	Hospital	Gastrectomy	58.1	13.1		60	42	18	Methylcobalamin (Oral) cyanocobalamin (IM)
12	2012	Tillemans	RCT		Netherland	Europe	Hospital	VB12 deficiency			>65	10			
13	2012	Saraswathy	RCT		South India	Asia	Hospital	VB12 deficiency	41.5			60			
14	2016	Parry-strong	RCT		New Zealand	Oceania	Hospital	Diabetes II, metformin	64.2	7.3		34	20	14	Sublingual: methylcobalamin; IM: hydroxocobalamin
15	2017	Metaxas	RCT	2017	Switzerland	Europe	Hospital	VB12 deficiency				37			Oral group (cyanocobalamin); intramuscular group (hydroxocobalamin)
16	2018	Schijns	RCT		Netherland	Europe	Hospital	Gastrectomy				50			Hydroxocobalamin (IM); methylcobalamin Oral
17	2018	Sezer	Prospective	2016	Turkey	Asia	Hospital	VB12 deficiency		0	18	142			Cyanocobalamin
18	2019	Bensky	Retrospective	2014–2016	Israel	Asia	Hospital	VB12 deficiency			>18	4,281			Cyanocobalamin
19	2020	Arıcan	Retrospective		Turkey	Asia	Hospital	Neurological symptoms			0–18	351			Cyanocobalamin
20	2020	Sanz-Cuesta	RCT		Spain	Europe	Hospital	VB12 deficiency	75.2	6.3		283	118	165	Cyanocobalamin
21	2020	Tuğba-kartal	Retrospective	2017–2019	Turkey	Asia	University hospital	VB12 deficiency			5–18	128			Cyanocobalamin
22	2021	Orhan kiliç	Retrospective	2017–2020	Turkey	Asia	University hospital	VB12 deficiency			0–3	158			Oral cyanocobalamin, sublingual methylcobalamin; intramuscular cyanocobalamin
23	2021	Ramos	Prospective	2017–2018	Brasil	South americ	Hospital	Gastrectomy	37.9	9,8	20–60	53			Cyanocobalamin
24	2022	Tandon	RCT	2015–2016	India	Asia	University hospital	VB12 deficiency			0.12–18	80			Methylcobalamin
25	2023	Korpeti	Prospective		Greece	Europe	Hospital					6			

The mean age of the enrolled participants was 53.8 (SD = 9). Gender-specific data were available in some studies, reporting 268 males and 288 females.

The included studies were conducted in 10 Countries across 5 continents (6 studies from Turkey; 4 from United States, 3 from Japan; 2 each from India, Israel and Netherlands and 1 each from Greece, Spain, Switzerland, Brazil, New Zealand and Korea). Seventeen studies were conducted in hospital settings, and the remaining 8 in university hospital settings.

Among the 25 studies included in the qualitative synthesis (16 in the meta-analysis), 15 focused specifically on serum cobalamin changes (reported by baseline and post-treatment means and standard deviations in pg/mL) and 10 reported homocysteine outcomes (means and standard deviations in mmol/L). Regarding the form of vitamin B12 used, cyanocobalamin was the most common (n = 14), followed by methylcobalamin (n = 6), hydroxocobalamin (n = 4), and combinations including adenosylcobalamin in isolated cases*.*

Additionally, 15 of the included studies focused on B12 supplementation, with a total of 5,191 participants (mean: 346 per study). In contrast, 10 studies investigated the relationship between B12 supplementation status and homocysteine levels, encompassing a total of 191 participants (mean: 19.1 per study).

### 3.2 Quality assessment and risk of bias

According to the Jadad scale for RCTs (n = 13) (Tanaka et al., 1981; Kuzminski et al., 1998; Bolaman et al., 2003; Sharabi et al., 2003; Yazaki et al., 2006; Castelli et al., 2011; Tillemans et al., 2014; Saraswathy et al., 2012; Parry-Strong et al., 2016; Metaxas et al., 2017; Schijns et al., 2018; Sanz-Cuesta et al., 2020; Tandon et al., 2022) the authors evaluated the quality of the clinical trial included in the qualitative synthesis, based on 5 items assessing the randomization process, blinding, and the dropout rate (i.e., the patients lost to follow-up). Quality scores ranged from 2 to 5 stars; additionally, a domain-specific assessment using the Cochrane Risk of Bias Tool (RoB 2) was also conducted and reported in Table 3 to provide a comprehensive appraisal. In the evaluation of RCT quality, 3 studies scored two points, indicating low-quality study and high risk of bias; 6 studies scored three points, and the remaining 4 scored five points, indicating medium and high-quality studies, respectively. Consistently, the risk of bias assessment, revealed a “moderate risk” for the first 6 studies scoring three points, and “high risk” for the 4 studies scoring five points.

**TABLE 3:** Jadad scale for randomised controlled trials.

Jadad scale for reporting randomised controlled trials							
Author	1) Is the study described as randomized?	2) Is the study described as double blind?	3) Is there a description of withdrawals and dropouts?	4) The method of randomisation is appropriate?	5) The method of blinding is appropriate?	Total score =	RoB score =
Tanaka et al. (1981)	1	0	0	1	0	2	High
Kuzminski et al. (1988)	1	0	0	1	0	2	High
Bolaman et al. (2003)	1	0	1	1	0	3	Moderate
Sharabi et al. (2003)	1	0	1	1	0	3	Moderate
Yazaki et al. (2006)	1	1	1	1	1	5	Low
Castelli et al. (2011)	1	1	1	1	1	5	Low
Tillemans et al. (2012)	1	0	1	0	0	2	High
Saraswathy et al. (2012)	1	1	1	1	1	5	Low
Parry-Strong (2016)	1	1	1	1	1	5	Low
Metaxas et al. (2017)	1	0	1	1	0	3	Moderate
Schijns et al. (2018)	1	0	1	1	0	3	Moderate
Sanz-Cuesta et al. (2020)	1	0	1	1	0	3	Moderate
Tandon et al. (2022)	1	0	1	1	0	3	Moderate

According to the Newcastle–Ottawa Scale (NOS) for case-control studies (n = 2) (Yamane et al., 1995; Adachi et al., 2000) and cohort studies (n = 10) (Altay et al., 1999; Roth and Orija, 2004; Kim et al., 2011; Sezer et al., 2018; Bensky et al., 2019; Arıcan et al., 2020; Tuğba-Kartal and Çağla-Mutlu, 2020; Orhan Kiliç et al., 2021; Ramos et al., 2021; Korpeti et al., 2023), the authors assessed study quality based on object selection, comparability and exposure. Risk of bias (RoB) scores, assessed using the NOS, ranged from 5 to 9 stars. The case-control studies both scored six, indicating high-quality studies with a moderate risk of bias (Table 4). Among the cohort studies, 2 scored three and five points, indicating low-quality studies with high and moderate risk of bias, respectively. 3 studies scored six, indicating medium-quality studies with a moderate risk of bias. 2 studies scored seven, indicating medium-quality studies with low risk of bias. The remaining 3 studies, scored eight and nine, indicating high-quality studies with low risk of bias (Table 5).

**TABLE 4:** Newcastle - Ottawa quality assessment scale for case-control studies.

Newcastle - Ottawa quality assessment scale case-control studies			
	Author	Yamane et al. (1995)	Adachi et al. (2000)
Selection: (Maximum 4 stars)	1) Is the case definition adequate?	*	*
	2) Representativeness of the cases		
	3) Selection of controls	*	*
	4) Definition of controls	*	*
Comparability: (Maximum 2 stars)	5) Comparability of cases and controls on the basis of the design or analysis	*	*
Outcome: (Maximum 3 stars)	6) Ascertainment of exposure	*	*
	7) Same method of ascertainment for cases and controls	*	*
	8) Non-response rate		
	Total score =	6	6
	RoB score =	Moderate	Moderate

**TABLE 5:** Newcastle - Ottawa quality assessment scale cohort studies.

Newcastle - Ottawa quality assessment scale cohort studies											
Author		Altay et al. (1999)	Roth and Orija. (2004)	Kim et al. (2011)	Sezer et al. (2018)	Bensky et al. (2019)	Arıcan et al. (2020)	Tuğba-Kartal and Çağla-Mutlu. (2020)	Orhan Kiliç et al. (2021)	Ramos et al. (2021)	Korpeti et al. (2023)
Selection: (Maximum 4 stars)	1) Representativeness of the exposed cohort		*	*	*	*	*	*	*		
	2) Selection of the non exposed cohort		*	*	*	*			*		
	3) Ascertainment of exposure	*	*	*	*	*	*	*	*	*	*
	4) Demonstration that outcome of interest was not present at start of study	*	*	*	*	*		*		*	
Comparability: (Maximum 2 stars)	5) Comparability of cohorts on the basis of the design or analysis	*	*	*	*	*	*	*	*	*	**
Outcome: (Maximum 3 stars)	6) Assessment of outcome **	*	**	**	**	**	**	*	**	**	*
	7) Was follow-up long enough for outcomes to occur	*	*	*		*	*	*	*	*	
	8) Adequacy of follow up of cohorts	*				*					
	Total score =	6	8	8	7	9	6	6	7	5	3
	RoB score	Moderate	Low	Low	Low	Low	Moderate	Moderate	Low	Moderate	High

The main sources of inconsistency in the NOS scale were sample representativeness and sample size. While all studies adequately ascertained exposure and ensured comparability between outcome groups by controlling for confounders, 42% (n = 5) did not provide adequate sample representativeness and size, and 17% (n = 2) did not report the non-response rate. In 67% of the studies (n = 8), the adequacy of follow-up of cohorts was not described.

The GRADE assessment showed moderate certainty of evidence for the increase in serum vitamin B12 levels and low certainty of evidence for homocysteine reduction, both across all studies and within RCT-only and observational-only subgroups. For observational studies reporting serum vitamin B12 levels, the certainty of evidence was upgraded from low to moderate due to the large and consistent magnitude of effect across studies, despite heterogeneity. High heterogeneity (I^2^ >80%) and concerns regarding imprecision and risk of bias were the main factors contributing to downgrading (Supplementary Table S3).

### 3.3 Changes in serum cobalamin by supplementation route and dosage

Across 35 comparisons, the mean percentage increase in serum cobalamin following supplementation was approximately 293%, confirming the overall effectiveness of vitamin B12 interventions (Supplementary Table S4).

When stratified by administration route, the mean percentage changes were:

- Intramuscular (IM): ∼307%
- Oral: ∼285%
- Sublingual: ∼199%

IM administration showed the highest average B12 increase, although oral and sublingual routes also yielded substantial improvements, supporting their use as effective and less invasive alternatives.

By dosage, the mean changes were:

- 500 mcg: ∼127%
- 750 mcg: ∼176% (single study)
- 1,000 mcg: ∼280%
- 1,500 mcg: ∼310%
- 2000 mcg: ∼260%
- 5,000 mcg: ∼62%

While 1,500 mcg of vitamin B12 showed the highest average increase, the effect plateaued or declined at higher doses, suggesting a possible saturation of absorption pathways. These findings support a personalised approach, where absorption efficiency may be more relevant than absolute dosage.

### 3.4 Changes in serum homocysteine by supplementation route and dosage

Analysis of 10 comparisons showed that vitamin B12 supplementation was associated with a mean reduction of −35.8% in serum homocysteine concentrations (Supplementary Table S5).

This confirms the well-documented role of cobalamin in lowering homocysteine levels in deficient or at-risk individuals.

When stratified by administration route, the average percentage reductions were:

- Intramuscular (IM): −48.3%
- Sublingual: −30.7%
- Oral: −30.3%

These results suggest that IM administration leads the most pronounced homocysteine reduction, however, sublingual and oral routes also demonstrate clinically relevant effects.

With respect to dosage, the average reductions were:

- 2000 mcg: −71.5% (single data point)
- 1,000 mcg: −31.8%

Although limited data are available for higher dosages, the findings support the efficacy of both oral and non-oral B12 formulations in modulating homocysteine levels. IM administration may provide the most robust response in clinical scenarios requiring rapid or substantial metabolic correction.

### 3.5 Meta-analysis

**FIGURE 2:**
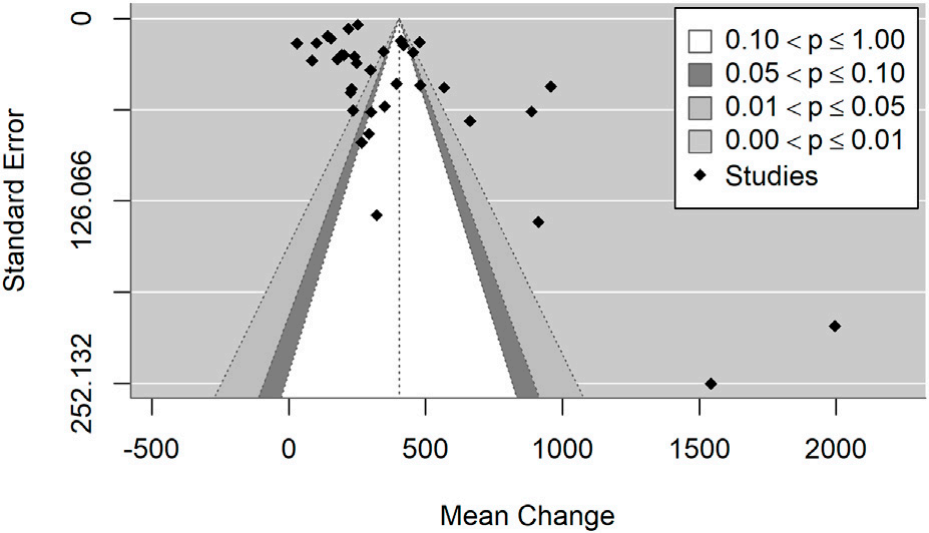
Funnel plot illustrating the mean change in cobalamin levels, indicating potential publication bias.

The included studies showed substantial heterogeneity (I^2^ = 99.6%, p < 0.001), suggesting considerable variability in study outcomes that cannot be attributed to chance alone. Egger’s test for funnel plot asymmetry (p < 0.001) indicates potential publication bias, which may influence the observed effect sizes (Figure 2). The prediction interval (−237.1, 1,042.3) reflects a wide range of potential true effects in future studies, highlighting the high heterogeneity of the included trials. Sensitivity analysis identified one influential study (Castelli et al., 2011), based on both DFFITS and Cook’s distance. This study reported by far the largest mean difference and very high variability. Excluding this study narrowed the prediction interval to (−83.2, 768.4), but only slightly reduced the heterogeneity (I^2^ = 99.2%). The increase in cobalamin levels remains significant (p < 0.001). There were no statistically significant differences in cobalamin levels between RCT and observational studies (p = 0.268).

The dependence of efficacy on administration route, underlying pathology, age and dosage was examined.

Some studies appear multiple times across the forest plots because they reported more than one independent comparison (for example, evaluating different administration routes or dosage subgroups within the same study population). In accordance with Cochrane and PRISMA recommendations, each comparison was treated as a separate data point in the meta-analysis to maintain data granularity and prevent loss of relevant information.

#### 3.5.1 Cobalamin levels by administration route

**FIGURE 3:**
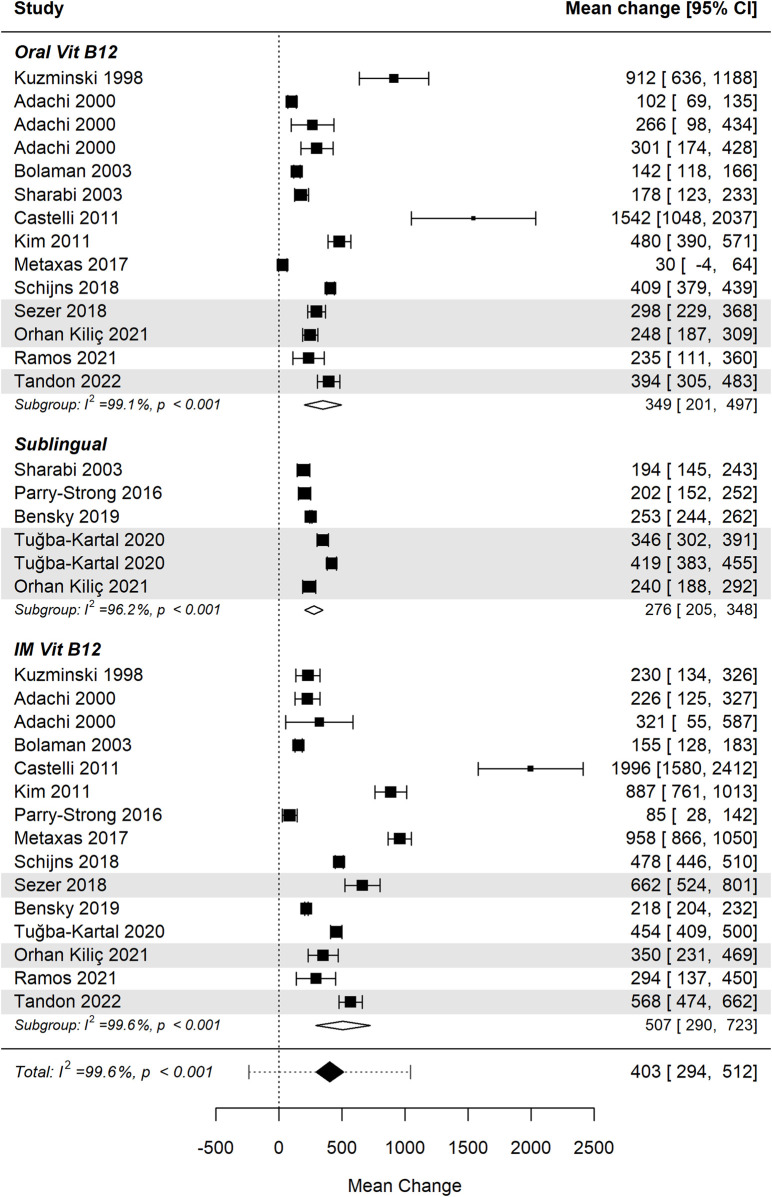
Cobalamin levels significantly increase following vitamin B12 administration. Differences between administration routes were not statistically significant. Studies involving children are shaded for visual differentiation.

Figure 3 presents the forest plot illustrating the effect of vitamin B12 supplementation across three administration routes: oral, sublingual, and intramuscular (IM) (Kuzminski et al., 1998; Adachi et al., 2000; Bolaman et al., 2003; Sharabi et al., 2003; Castelli et al., 2011; Kim et al., 2011; Parry-Strong et al., 2016; Metaxas et al., 2017; Schijns et al., 2018; Sezer et al., 2018; Bensky et al., 2019; Tuğba-Kartal and Çağla-Mutlu, 2020; Orhan Kiliç et al., 2021; Ramos et al., 2021; Tandon et al., 2022). The pooled analysis demonstrates a statistically significant increase in cobalamin levels following B12 administration (p < 0.001), indicating a substantial effect size across all groups. Positive values reflect an overall increase in serum B12 concentration post-administration. Importantly, the differences between administration routes were not statistically significant (p = 0.270), suggesting comparable efficacy among oral, sublingual, and intramuscular B12 administration.

### 3.6 Cobalamin levels by underlying pathology

**FIGURE 4:**
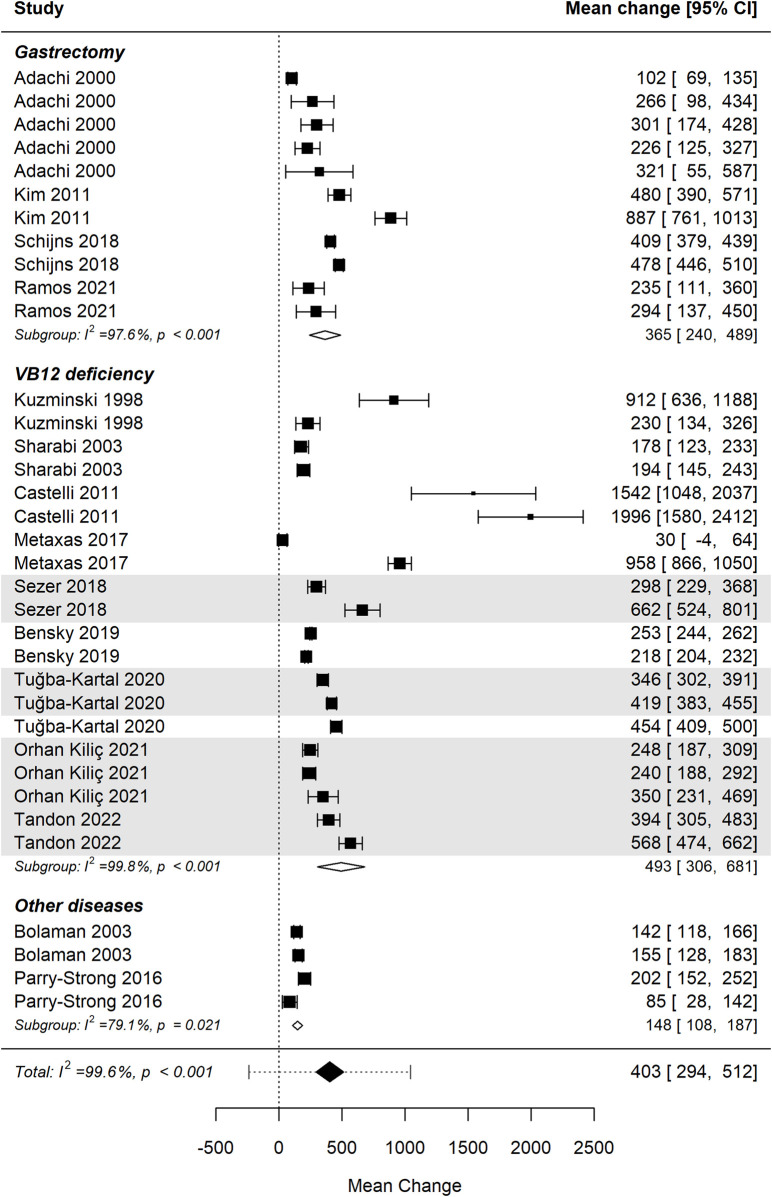
Differences in mean change in cobalamin levels between pathology groups were not statistically significant. Studies involving children are shaded for visual differentiation.

Figure 4 presents the forest plot illustrating the effect of vitamin B12 administration stratified by underlying pathology: gastrectomy, vitamin B12 deficiency of unspecified etiology, and other diseases (Kuzminski et al., 1998; Adachi et al., 2000; Bolaman et al., 2003; Sharabi et al., 2003; Castelli et al., 2011; Kim et al., 2011; Parry-Strong et al., 2016; Metaxas et al., 2017; Schijns et al., 2018; Sezer et al., 2018; Bensky et al., 2019; Tuğba-Kartal and Çağla-Mutlu, 2020; Orhan Kiliç et al., 2021; Ramos et al., 2021; Tandon et al., 2022). The pooled analysis demonstrates a significant increase in cobalamin levels across all subgroups. However, the difference between pathology groups was not statistically significant (p = 0.132), suggesting a comparable response to B12 supplementation across clinical conditions: i) Gastrectomy: this subgroup includes patients who have undergone partial or total gastrectomy, a condition known to impair intrinsic factor-mediated absorption of vitamin B12. The pooled effect estimate indicates a substantial increase in B12 levels following supplementation; ii) Vitamin B12 Deficiency (unspecified etiology): this category includes individuals diagnosed with B12 deficiency without a clearly defined cause. The effect size within this group is similar to that of gastrectomy, highlighting the broad applicability of B12 supplementation across different deficiency states; iii) Other Diseases: this smaller subgroup includes conditions in which B12 deficiency may be secondary to disease-related malabsorption or altered metabolism. The observed heterogeneity in effect size suggests variability in response within this group. These findings underscore the efficacy of B12 supplementation across a range of pathological conditions while emphasizing the need for further research to better define disease-specific responses and optimal dosing strategies.

#### 3.6.1 Cobalamin levels by age group

**FIGURE 5:**
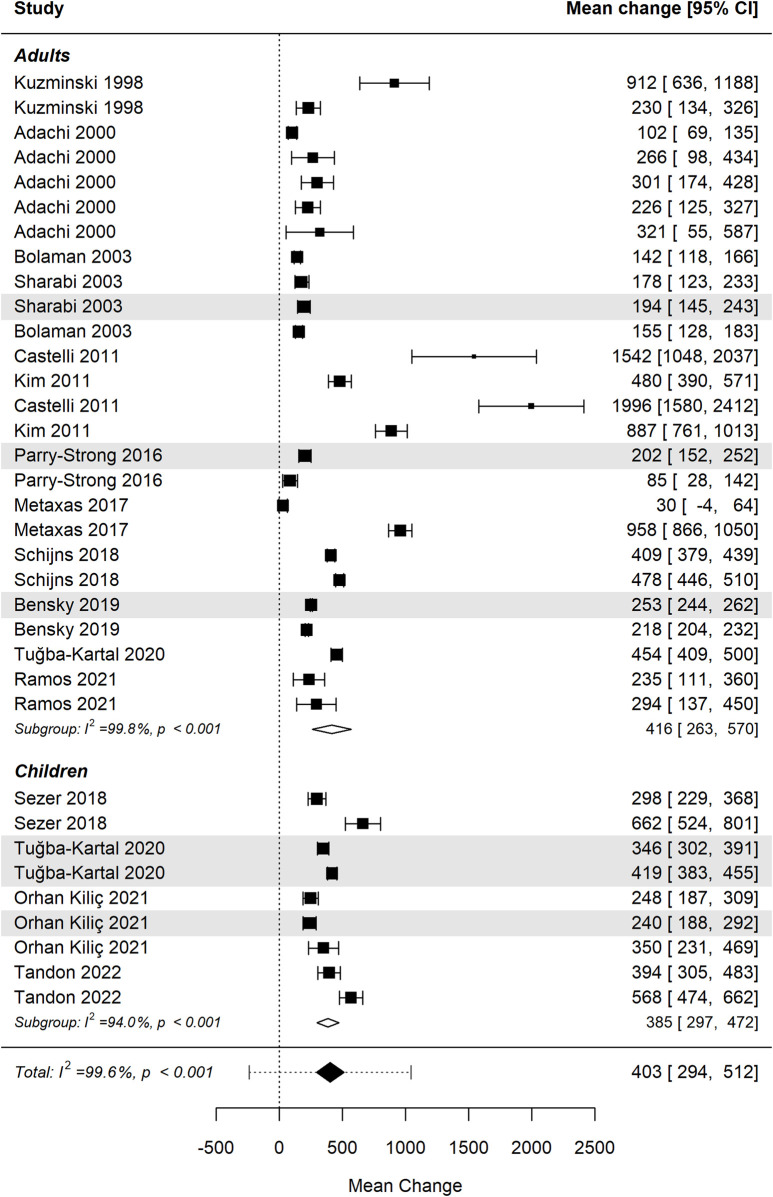
Differences in mean change in cobalamin levels between age groups were not statistically significant. Studies using the sublingual route are shaded for visual differentiation.

Figure 5 presents the forest plot illustrating the effect of vitamin B12 administration stratified by age group (adults vs. children) (Kuzminski et al., 1998; Adachi et al., 2000; Bolaman et al., 2003; Sharabi et al., 2003; Castelli et al., 2011; Kim et al., 2011; Parry-strong et al., 2016; Metaxas et al., 2017; Schijns et al., 2018; Sezer et al., 2018; Bensky et al., 2019; Tuğba-Kartal and Çağla-Mutlu, 2020; Orhan Kiliç et al., 2021; Ramos et al., 2021; Tandon et al., 2022). The pooled analysis demonstrates a significant overall increase in cobalamin levels following B12 supplementation in both subgroups: i) Adults: the effect size in adults shows substantial heterogeneity, reflecting variability in response due to factors such as baseline B12 status, absorption capacity, and deficiency severity; ii) Children (age range 0–15 years): studies involving pediatric populations show similarly significant increase in cobalamin levels, with effect sizes comparable to those observed in adults. Studies using the sublingual route are shaded for visual differentiation.

Despite differences in effect sizes across individual studies, the overall difference between age groups was not statistically significant (p = 0.902), indicating indicating similar efficacy of B12 supplementation in both adults and children. Further research is warranted to investigate influences such as baseline deficiency status, absorption efficiency, and dosing regimens across age groups.

#### 3.6.2 Cobalamin levels by dosage

**FIGURE 6:**
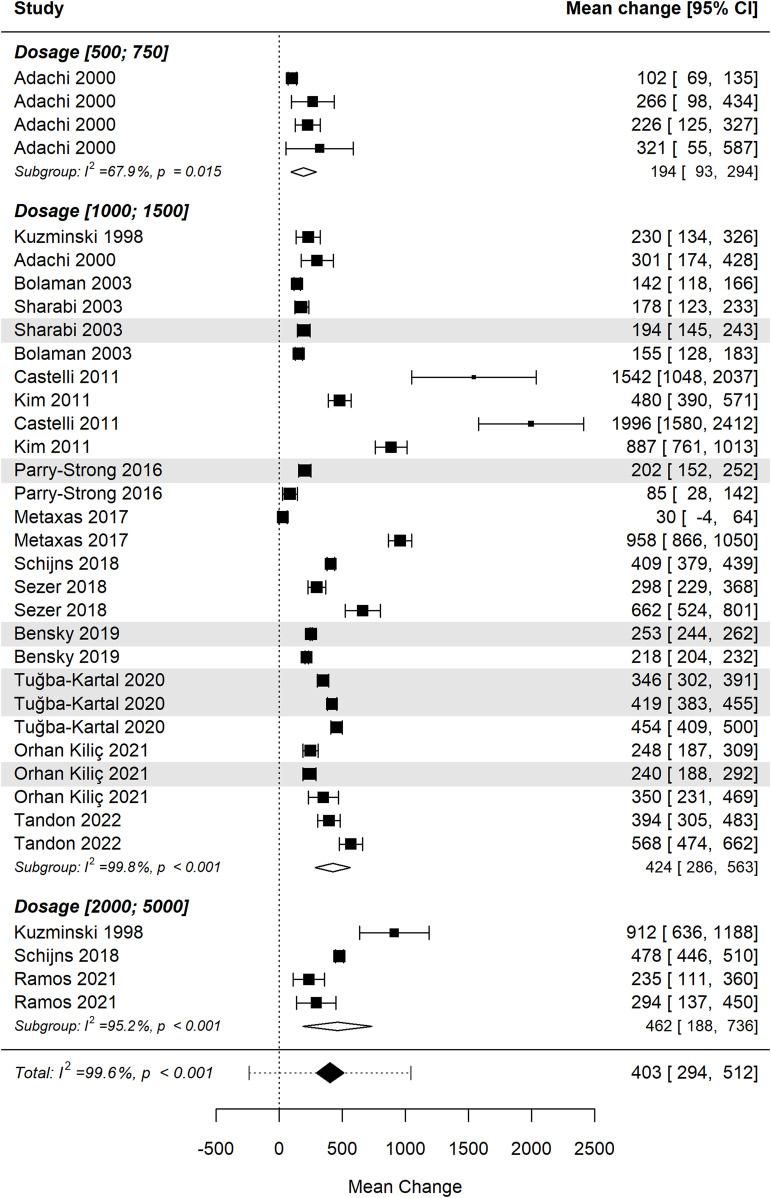
Differences in mean change in cobalamin levels between dosage groups were not statistically significant. Studies using the sublingual route are shaded for visual differentiation.

Figure 6 presents the forest plot illustrating the effect of vitamin B12 supplementation stratified by dosage range: 500–750 µg, 1000–1500 µg, and 2000–5000 µg (Kuzminski et al., 1998; Adachi et al., 2000; Bolaman et al., 2003; Sharabi et al., 2003; Castelli et al., 2011; Kim et al., 2011; Parry-Strong et al., 2016; Metaxas et al., 2017; Schijns et al., 2018; Sezer et al., 2018; Bensky et al., 2019; Tuğba-Kartal and Çağla-Mutlu, 2020; Orhan Kiliç et al., 2021; Ramos et al., 2021; Tandon et al., 2022). The pooled analysis indicates a significant increase in cobalamin levels across all dosage groups following supplementation. However, the differences between dosage groups were not statistically significant (p = 0.485), suggesting that higher doses do not necessarily yield greater efficacy in raising serum B12 levels: i) Low-dose group (500–750 µg): this subgroup shows moderate heterogeneity (I^2^ = 67.9%, p = 0.015), indicating some variability in response, though the overall effect size remains positive; ii) Moderate-dose group (1000–1500 µg): the largest subgroup in this analysis, demonstrating substantial heterogeneity (I^2^ = 99.8%, p < 0.001) with considerable variability in effect size across studies; iii) High-dose group (2000–5000 µg): this subgroup shows significant increases in cobalamin levels, although with continued high heterogeneity (I^2^ = 95.2%, p < 0.001), suggesting a variable response even at higher doses.

Despite differences in absolute mean changes, the lack of a statistically significant dose-response relationship implies that factors beyond dosage—such as baseline B12 deficiency, absorption efficiency, and individual metabolic differences—may play a more critical role in determining serum B12 increases. Further studies are needed to determine optimal dosing strategies, particularly in populations with varying deficiency states, absorption impairments, and treatment durations.

#### 3.6.3 Homocysteine levels by administration route

**FIGURE 7:**
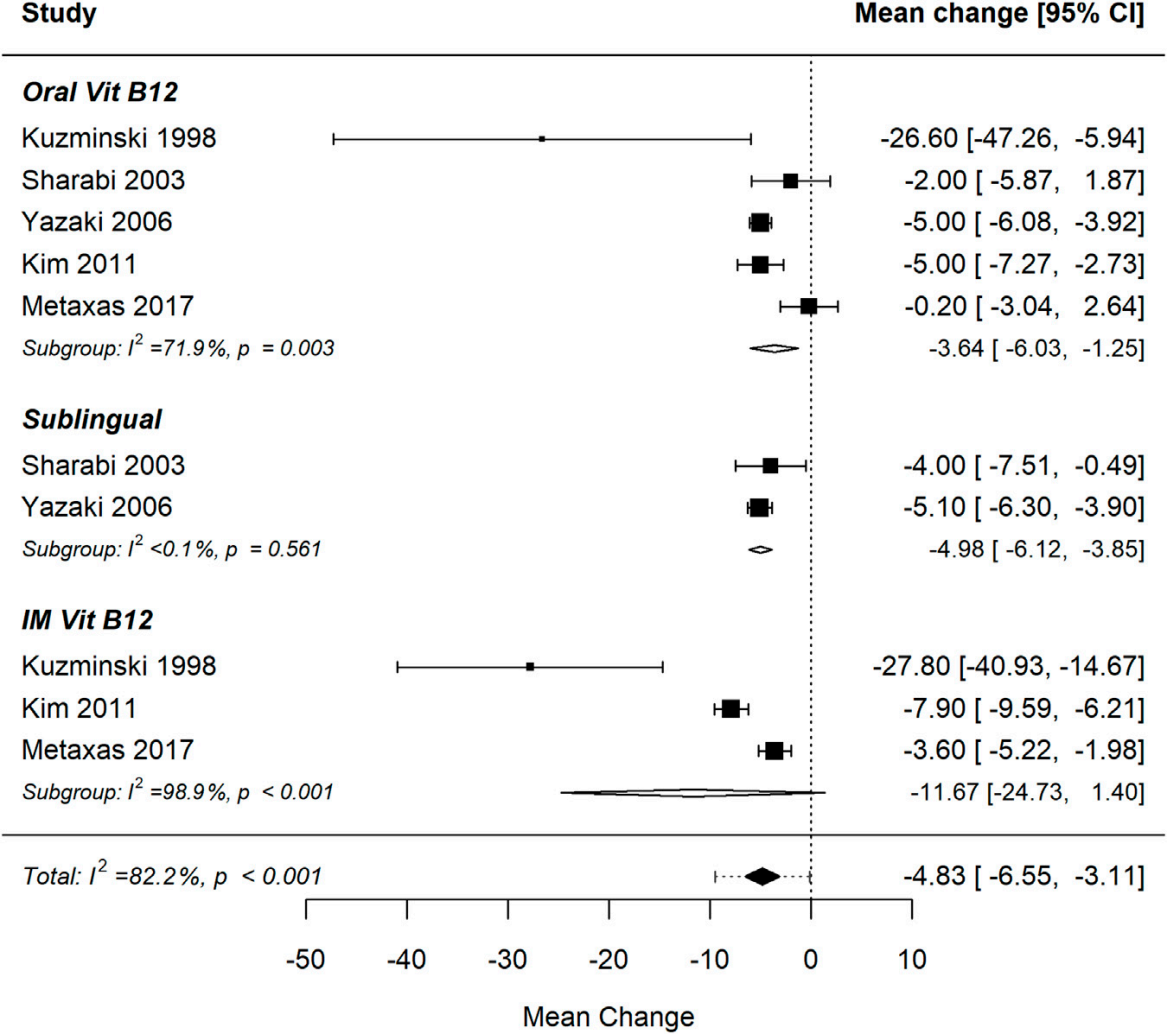
Homocysteine levels significantly decrease following vitamin B12 administration. Differences between administration routes were not statistically significant.

**FIGURE 8:**
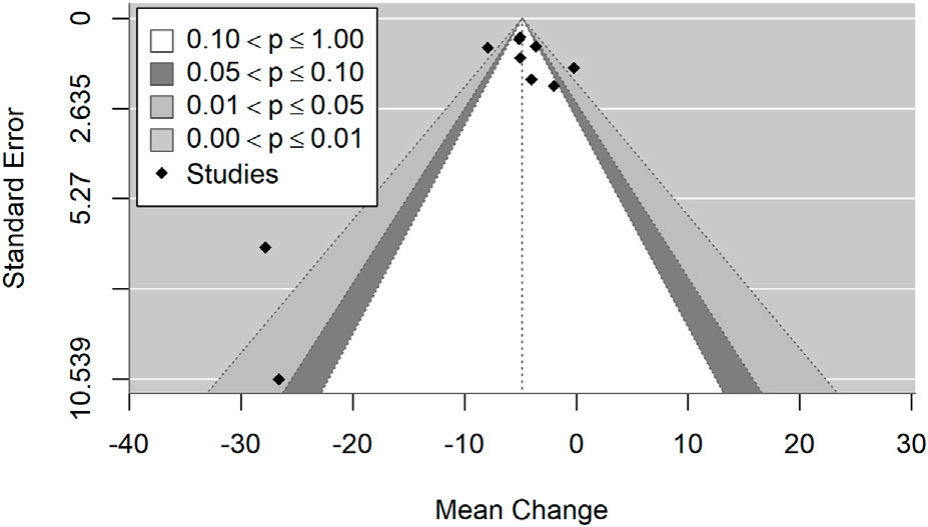
Funnel plot illustrating the mean change in homocysteine levels, indicating potential publication bias.

Figures 7, 8 present the forest plot and funnel plot illustrating the effect of vitamin B12 supplementation on homocysteine (Hcy) levels, stratified by administration route (oral, sublingual, and intramuscular) (Kuzminski et al., 1998; Sharabi et al., 2003; Yazaki et al., 2006). The pooled analysis shows a statistically significant reduction in homocysteine levels following B12 administration (p < 0.001), with a negative effect size indicating a decrease in serum Hcy concentrations post-treatment. This finding is consistent across all administration routes, confirming the role of vitamin B12 in homocysteine metabolism through its cofactor role in one-carbon metabolism. There were no statistically significant differences in homocysteine levels between RCT and observational studies (p = 0.139). Substantial heterogeneity was observed (I^2^ = 82.2%, p < 0.001), indicating a high degree of variability among studies that cannot be attributed to chance alone. Egger’s test for funnel plot asymmetry (p = 0.003) suggests potential publication bias, indicating that smaller studies with less significant results may be underrepresented. The prediction interval (−9.5, −0.2) suggests that future studies are likely to observe a reduction in homocysteine levels, but with some variability in effect size. Sensitivity analysis identified one influential study (Kuzminski et al., 1998), based on both DFFITS and Cook’s distance. This study reported the largest mean difference and very high variability. Excluding it only slightly narrowed the prediction interval to (−8.4, −0.4) and reduced the heterogeneity (I^2^ = 81.0%). The reduction in homocysteine levels remains significant (p < 0.001).

The difference in homocysteine reduction between administration routes was not statistically significant (p = 0.485), suggesting that oral, sublingual, and IM administration are similarly effective in lowering Hcy levels. These results confirm the efficacy of vitamin B12 supplementation in reducing homocysteine, an important biomarker linked to cardiovascular risk, neurological and metabolic disorders. However, given the observed heterogeneity and potential publication bias, further well-designed, large-scale randomised controlled trials (RCTs) are warranted to refine optimal dosing and administration strategies.

To further explore the relationship between administration route and dosage, we generated a supplementary plot combining these two variables (Supplementary Figure S1). This visualization illustrates comparable increases in serum cobalamin levels across all routes and dosage groups, with overlapping standard deviations, reinforcing the absence of a clear dose-dependent effect.

No statistically significant differences were observed between administration routes or dosage groups (p > 0.05), supporting the conclusion of comparable efficacy and the lack of a clear dose–response relationship.

## 4 Discussion

This systematic review and meta-analysis provide a comparative evaluation of oral, sublingual, and intramuscular (IM) vitamin B12 supplementation in the management of B12 deficiency. Our findings indicate that all three administration routes effectively increase serum B12 levels, with no statistically significant differences among them. These results suggest that sublingual and oral B12 supplementation may serve as viable alternatives to IM injections, particularly in clinical settings where frequent intramuscular administration is impractical or resource intensive.

Despite comparable efficacy, route-specific considerations remain relevant. IM administration is the most invasive and costly, requiring healthcare visits, trained personnel, and procedural infrastructure. While it ensures direct systemic absorption, it is less patient-friendly and may result in reduced treatment adherence. Oral B12 supplementation is more accessible and cost-effective but relies on gastric intrinsic factor for absorption, rendering it unsuitable for individuals with gastric disorders or malabsorption syndromes. Sublingual administration, which bypasses the gastrointestinal tract, appears to be equally effective as IM injections while being less invasive and more cost-efficient—particularly in patients with atrophic gastritis, post-gastrectomy status, or conditions that impair intrinsic factor-mediated absorption, where long-term intervention is often required.

Interestingly, much of the research has been based on doses far exceeding the recommended daily intake, both in individuals with pathological conditions and in healthy subjects. Despite the absence of a statistically significant dose–response relationship observed in this analysis, clinical decision-making should consider that absorption efficiency—rather than absolute dosage—may be the limiting factor. This is particularly relevant for oral administration, which is subject to intrinsic factor saturation and receptor limitations in the ileum (Paul and Brady, 2017; Chanarin, 2000). Some pharmacokinetic studies suggest that only around 1.5–2.5 μg of B12 is absorbed from food per meal, while approximately 5% of a 25 μg dose and only 1% of a 1,000 μg oral dose is effectively absorbed (Devi et al., 2020; Carmel, 2008). These findings support the rationale for frequent, low-dose supplementation strategies in healthy individuals, while also emphasising the need for personalised approaches in those with malabsorption syndromes (Bor et al., 2010).

Moreover, our results highlight the essential role of vitamin B12 in homocysteine metabolism, as supplementation was associated with a significant reduction in serum homocysteine levels—an important biomarker associated with cardiovascular and neurodegenerative diseases. However, substantial heterogeneity among studies was observed, likely attributable to differences in dosage, baseline B12 status, and population characteristics. The presence of funnel plot asymmetry also suggests potential publication bias, which warrants further scrutiny. These observations reinforce the need for well-designed randomised controlled trials (RCTs) to validate the findings and expand the evidence base.

From a public health perspective, our findings support the implementation of non-invasive vitamin B12 supplementation strategies. Given the lifelong need for B12 therapy in deficiency-prone populations, the selection of the most patient-friendly and economically sustainable option is critical. Sublingual administration, offering a balance of efficacy, accessibility, and compliance, emerges as a promising approach for routine clinical use. Future research should prioritise long-term outcomes in high-risk groups, comparisons of physiological versus pharmacological dosages in healthy subjects, cost-effectiveness analyses, and adherence monitoring to inform clinical guidelines for the management and prevention of B12 deficiency.

### 4.1 Limitations and implications for practice

This meta-analysis has several limitations that should be considered when interpreting the findings. The substantial heterogeneity observed across studies (I^2^ > 80%) reflects differences in populations, dosages, supplementation duration, and analytical methods, which may have influenced the pooled estimates. Additionally, the presence of publication bias suggested by Egger’s test and funnel plot asymmetry indicates that smaller studies with non-significant results might be underrepresented. The methodological quality of the included studies, as assessed through the Jadad and Newcastle–Ottawa scales, varied from moderate to high, while the GRADE assessment indicated moderate certainty of evidence for serum vitamin B12 increase and low certainty for homocysteine reduction. These factors collectively suggest that the conclusions, while robust, should be interpreted with caution in clinical decision-making. Future research should focus on large-scale, well-controlled randomized trials with standardized dosing protocols, clearly defined deficiency thresholds, and long-term follow-up to strengthen the evidence base and guide clinical practice.

## 5 Conclusion

This systematic review and meta-analysis indicate that sublingual and oral vitamin B12 administration provide efficacy comparable to intramuscular injection in improving serum cobalamin levels and reducing hyperhomocysteinaemia. Given their non-invasive nature and similar clinical performance, these routes may represent suitable alternatives to intramuscular therapy, particularly in settings requiring long-term management. Future research should investigate long-term outcomes in high-risk populations, direct comparisons between physiological and pharmacological dosages, cost-effectiveness analyses, and adherence metrics to inform optimal strategies for the prevention and management of vitamin B12 deficiency.

## Data Availability

The original contributions presented in the study are included in the article/Supplementary Material, further inquiries can be directed to the corresponding author.
