# Supplementary material for: Efficacy of sublingual and oral vitamin B12 versus intramuscular administration: insights from a systematic review and meta-analysis
Source: Front Pharmacol. 2025 Dec 19;16:1602976. doi: 10.3389/fphar.2025.1602976 (PMC12757266; doi:10.3389/fphar.2025.1602976)
Supplement: Supplementary file 1 [file Supplementaryfile1.docx]

Supplementary Material

# Supplementary Tables

Table S1. Prisma 2020 check list

| **Section and Topic** | **Item #** | **Checklist item** | **Location where item is reported** |
| --- | --- | --- | --- |
| **TITLE** | | |  |
| Title | 1 | Identify the report as a systematic review. | Title |
| **ABSTRACT** | | |  |
| Abstract | 2 | See the PRISMA 2020 for Abstracts checklist. |  |
| **INTRODUCTION** | | |  |
| Rationale | 3 | Describe the rationale for the review in the context of existing knowledge. | Introduction |
| Objectives | 4 | Provide an explicit statement of the objective(s) or question(s) the review addresses. | Introduction |
| **METHODS** | | |  |
| Eligibility criteria | 5 | Specify the inclusion and exclusion criteria for the review and how studies were grouped for the syntheses. | Materials and methods |
| Information sources | 6 | Specify all databases, registers, websites, organisations, reference lists and other sources searched or consulted to identify studies. Specify the date when each source was last searched or consulted. | Materials and methods |
| Search strategy | 7 | Present the full search strategies for all databases, registers and websites, including any filters and limits used. | Materials and methods |
| Selection process | 8 | Specify the methods used to decide whether a study met the inclusion criteria of the review, including how many reviewers screened each record and each report retrieved, whether they worked independently, and if applicable, details of automation tools used in the process. | Materials and methods |
| Data collection process | 9 | Specify the methods used to collect data from reports, including how many reviewers collected data from each report, whether they worked independently, any processes for obtaining or confirming data from study investigators, and if applicable, details of automation tools used in the process. | Materials and methods |
| Data items | 10a | List and define all outcomes for which data were sought. Specify whether all results that were compatible with each outcome domain in each study were sought (e.g. for all measures, time points, analyses), and if not, the methods used to decide which results to collect. | Materials and methods |
|  | 10b | List and define all other variables for which data were sought (e.g. participant and intervention characteristics, funding sources). Describe any assumptions made about any missing or unclear information. | Materials and methods |
| Study risk of bias assessment | 11 | Specify the methods used to assess risk of bias in the included studies, including details of the tool(s) used, how many reviewers assessed each study and whether they worked independently, and if applicable, details of automation tools used in the process. | Materials and methods |
| Effect measures | 12 | Specify for each outcome the effect measure(s) (e.g. risk ratio, mean difference) used in the synthesis or presentation of results. | Materials and methods |
| Synthesis methods | 13a | Describe the processes used to decide which studies were eligible for each synthesis (e.g. tabulating the study intervention characteristics and comparing against the planned groups for each synthesis (item #5)). | Materials and methods |
|  | 13b | Describe any methods required to prepare the data for presentation or synthesis, such as handling of missing summary statistics, or data conversions. | Materials and methods |
|  | 13c | Describe any methods used to tabulate or visually display results of individual studies and syntheses. | Materials and methods |
|  | 13d | Describe any methods used to synthesize results and provide a rationale for the choice(s). If meta-analysis was performed, describe the model(s), method(s) to identify the presence and extent of statistical heterogeneity, and software package(s) used. | Materials and methods |
|  | 13e | Describe any methods used to explore possible causes of heterogeneity among study results (e.g. subgroup analysis, meta-regression). | Materials and methods |
|  | 13f | Describe any sensitivity analyses conducted to assess robustness of the synthesized results. | Materials and methods |
| Reporting bias assessment | 14 | Describe any methods used to assess risk of bias due to missing results in a synthesis (arising from reporting biases). | Materials and methods |
| Certainty assessment | 15 | Describe any methods used to assess certainty (or confidence) in the body of evidence for an outcome. | Materials and methods |
| **RESULTS** | | |  |
| Study selection | 16a | Describe the results of the search and selection process, from the number of records identified in the search to the number of studies included in the review, ideally using a flow diagram. | Results |
|  | 16b | Cite studies that might appear to meet the inclusion criteria, but which were excluded, and explain why they were excluded. | Results |
| Study characteristics | 17 | Cite each included study and present its characteristics. | Results |
| Risk of bias in studies | 18 | Present assessments of risk of bias for each included study. | Results |
| Results of individual studies | 19 | For all outcomes, present, for each study: (a) summary statistics for each group (where appropriate) and (b) an effect estimate and its precision (e.g. confidence/credible interval), ideally using structured tables or plots. | Results |
| Results of syntheses | 20a | For each synthesis, briefly summarise the characteristics and risk of bias among contributing studies. | Results |
|  | 20b | Present results of all statistical syntheses conducted. If meta-analysis was done, present for each the summary estimate and its precision (e.g. confidence/credible interval) and measures of statistical heterogeneity. If comparing groups, describe the direction of the effect. | Results |
|  | 20c | Present results of all investigations of possible causes of heterogeneity among study results. | Results |
|  | 20d | Present results of all sensitivity analyses conducted to assess the robustness of the synthesized results. | Results |
| Reporting biases | 21 | Present assessments of risk of bias due to missing results (arising from reporting biases) for each synthesis assessed. | Results |
| Certainty of evidence | 22 | Present assessments of certainty (or confidence) in the body of evidence for each outcome assessed. | Results |
| **DISCUSSION** | | |  |
| Discussion | 23a | Provide a general interpretation of the results in the context of other evidence. | Discussion |
|  | 23b | Discuss any limitations of the evidence included in the review. | Discussion |
|  | 23c | Discuss any limitations of the review processes used. | Discussion |
|  | 23d | Discuss implications of the results for practice, policy, and future research. | Discussion |
| **OTHER INFORMATION** | | |  |
| Registration and protocol | 24a | Provide registration information for the review, including register name and registration number, or state that the review was not registered. | Materials and methods |
|  | 24b | Indicate where the review protocol can be accessed, or state that a protocol was not prepared. | Materials and methods |
|  | 24c | Describe and explain any amendments to information provided at registration or in the protocol. | Materials and methods |
| Support | 25 | Describe sources of financial or non-financial support for the review, and the role of the funders or sponsors in the review. | Conclusions |
| Competing interests | 26 | Declare any competing interests of review authors. | Conclusions |
| Availability of data, code and other materials | 27 | Report which of the following are publicly available and where they can be found: template data collection forms; data extracted from included studies; data used for all analyses; analytic code; any other materials used in the review. | Supplementary content |

Table S2. Search strings used for each database

| PubMed | *("administration"[Title/Abstract] OR "implementation"[Title/Abstract] OR "supplementation"[Title/abstract] OR "delivery"[Title/abstract] OR "treatment"[Title/Abstract]) AND ("transmucosal"[Title/Abstract] OR "mucosal"[Title/Abstract] OR "oral"[Title/Abstract] OR "sublingual"[Title/Abstract] OR "buccal"[Title/Abstract]) AND ("vitamin b 12"[MeSH Terms] OR "vitamin b 12"[All Fields] OR ("vitamin"[All Fields] AND "b12"[All Fields]) OR "vitamin b12"[All Fields] OR "cobalamin"[All Fields] OR "cyanocobalamin"[All Fields] OR "hydroxocobalamin"[All Fields] OR "methylcobalamin"[All Fields] OR "Cyano-B12"[All Fields] OR "Hydroxo-B12"[All Fields])* |
| --- | --- |
| Embase | *(‘administration’:ti,ab OR ‘implementation’:ti,ab OR ‘supplementation’:ti,ab OR ‘delivery’:ti,ab OR ‘treatment’:ti,ab ) AND (‘transmucosal’:ti,ab OR ‘mucosal’:ti,ab OR ‘oral’:ti,ab OR ‘syblingual’:ti,ab OR ‘buccal’:ti,ab) AND ('vitamin b group':ti,ab OR'cyanocobalamin':ti,ab OR 'cobalamin':ti,ab OR 'hydroxocobalamin':ti,ab OR 'mecobalamin':ti,ab)* |
| Scopus | *( TITLE-ABS-KEY ( administration ) OR TITLE-ABS-KEY ( implementation ) OR TITLE-ABS-KEY ( supplementation ) OR TITLE-ABS-KEY ( delivery ) OR TITLE-ABS-KEY ( treatment ) ) AND ( TITLE-ABS-KEY ( transmucosal ) OR TITLE-ABS-KEY ( mucosal ) OR TITLE-ABS-KEY ( sublingual ) OR TITLE-ABS-KEY ( buccal ) ) AND ( TITLE-ABS-KEY ( "vitamin b12" ) OR TITLE-ABS-KEY ( "vitamin b 12" ) OR TITLE-ABS-KEY ( "cobalamin" ) OR TITLE-ABS-KEY ( "cyanocobalamin" ) OR TITLE-ABS-KEY ( "hydroxocobalamin" ) OR TITLE-ABS-KEY ( "methylcobalamin" ) OR TITLE-ABS-KEY ( "Cyano-B12" ) OR TITLE-ABS-KEY ( "Hydroxo-B12" ) )* |

Table S3. Changes in percentage in Serum Cobalamin

| **Study** | **Type of sample** | **Health status** | **Route** | **Dosage** | **Mean1** | **Mean2** | **Change (%)** |
| --- | --- | --- | --- | --- | --- | --- | --- |
| Kuzminski 1998 | Adults | VB12 deficiency | Oral Vit B12 | 2000 | 93 | 1005 | 980,6 |
| Kuzminski 1998 | Adults | VB12 deficiency | IM Vit B12 | 1000 | 95 | 325 | 242,1 |
| Adachi 2000 | Adults | Gastrectomy | Oral Vit B12 | 500 | 132 | 234 | 77,3 |
| Adachi 2000 | Adults | Gastrectomy | Oral Vit B12 | 750 | 151 | 417 | 176,2 |
| Adachi 2000 | Adults | Gastrectomy | Oral Vit B12 | 1500 | 112 | 413 | 268,8 |
| Adachi 2000 | Adults | Gastrectomy | IM Vit B12 | 500 | 102 | 328 | 221,6 |
| Adachi 2000 | Adults | Gastrectomy | IM Vit B12 | 500 | 127 | 448 | 252,8 |
| Bolaman 2003 | Adults | Other diseases | Oral Vit B12 | 1000 | 72 | 213,8 | 196,9 |
| Sharabi 2003 | Adults | VB12 deficiency | Oral Vit B12 | 1000 | 108 | 286 | 164,8 |
| Sharabi 2003 | Adults | VB12 deficiency | Sublingual | 1000 | 94 | 288 | 206,4 |
| Bolaman 2003 | Adults | Other diseases | IM Vit B12 | 1000 | 70,2 | 225,5 | 221,2 |
| Castelli 2011 | Adults | VB12 deficiency | Oral Vit B12 | 1000 | 285,5 | 1828 | 540,3 |
| Kim 2011 | Adults | Gastrectomy | Oral Vit B12 | 1000 | 169,8 | 650,3 | 283,0 |
| Castelli 2011 | Adults | VB12 deficiency | IM Vit B12 | 1000 | 262 | 2258 | 761,8 |
| Kim 2011 | Adults | Gastrectomy | IM Vit B12 | 1000 | 160 | 1047,2 | 554,5 |
| Parry-Strong 2016 | Adults | Other diseases | Sublingual | 1000 | 170,2 | 372,1 | 118,6 |
| Parry-Strong 2016 | Adults | Other diseases | IM Vit B12 | 1000 | 166,7 | 251,7 | 51,0 |
| Metaxas 2017 | Adults | VB12 deficiency | Oral Vit B12 | 1000 | 158 | 188 | 19,0 |
| Metaxas 2017 | Adults | VB12 deficiency | IM Vit B12 | 1000 | 164 | 1122 | 584,1 |
| Schijns 2018 | Adults | Gastrectomy | Oral Vit B12 | 1000 | 167 | 576 | 244,9 |
| Sezer 2018 | Children | VB12 deficiency | Oral Vit B12 | 1000 | 183,5 | 482 | 162,7 |
| Schijns 2018 | Adults | Gastrectomy | IM Vit B12 | 2000 | 175 | 653 | 273,1 |
| Sezer 2018 | Children | VB12 deficiency | IM Vit B12 | 1000 | 175,5 | 838 | 377,5 |
| Bensky 2019 | Adults | VB12 deficiency | Sublingual | 1000 | 298 | 551 | 84,9 |
| Bensky 2019 | Adults | VB12 deficiency | IM Vit B12 | 1000 | 234 | 452 | 93,2 |
| Tuğba-Kartal 2020 | Children | VB12 deficiency | Sublingual | 1000 | 137,2 | 483,4 | 252,3 |
| Tuğba-Kartal 2020 | Children | VB12 deficiency | Sublingual | 1000 | 146,7 | 565,5 | 285,5 |
| Tuğba-Kartal 2020 | Adults | VB12 deficiency | IM Vit B12 | 1000 | 147,5 | 602 | 308,1 |
| Orhan Kiliç 2021 | Children | VB12 deficiency | Oral Vit B12 | 1000 | 201,1 | 449,2 | 123,4 |
| Ramos 2021 | Adults | Gastrectomy | Oral Vit B12 | 5000 | 454,1 | 689,4 | 51,8 |
| Orhan Kiliç 2021 | Children | VB12 deficiency | Sublingual | 1000 | 187 | 427,1 | 128,4 |
| Orhan Kiliç 2021 | Children | VB12 deficiency | IM Vit B12 | 1000 | 176,1 | 526,1 | 198,8 |
| Ramos 2021 | Adults | Gastrectomy | IM Vit B12 | 5000 | 419,2 | 712,9 | 70,1 |
| Tandon 2022 | Children | VB12 deficiency | Oral Vit B12 | 1500 | 112 | 506 | 351,8 |
| Tandon 2022 | Children | VB12 deficiency | IM Vit B12 | 1000 | 85 | 653 | 668,2 |

Table S4. Changes in percentage in Serum Homocysteine

| **Study** | **Type of sample** | **Health status** | **Route** | **Dosage** | **Mean1** | **Mean2** | **Change (%)** |
| --- | --- | --- | --- | --- | --- | --- | --- |
| Kuzminski 1998 | Adults | VB12 deficiency | Oral Vit B12 | 2000 | 37,2 | 10,6 | -71,5 |
| Kuzminski 1998 | Adults | VB12 deficiency | IM Vit B12 | 1000 | 40 | 12,2 | -69,5 |
| Sharabi 2003 | Adults | VB12 deficiency | Oral Vit B12 | 1000 | 15 | 13 | -13,3 |
| Sharabi 2003 | Adults | VB12 deficiency | Sublingual | 1000 | 16 | 12 | -25,0 |
| Yazaki 2006 | Adults | VB12 deficiency | Oral Vit B12 | 1000 | 13,7 | 8,7 | -36,5 |
| Yazaki 2006 | Adults | VB12 deficiency | Sublingual | 1000 | 14 | 8,9 | -36,4 |
| Kim 2011 | Adults | Gastrectomy | Oral Vit B12 | 1000 | 17,5 | 12,5 | -28,6 |
| Kim 2011 | Adults | Gastrectomy | IM Vit B12 | 1000 | 16,5 | 8,6 | -47,9 |
| Metaxas 2017 | Adults | VB12 deficiency | Oral Vit B12 | 1000 | 14,8 | 14,6 | -1,4 |
| Metaxas 2017 | Adults | VB12 deficiency | IM Vit B12 | 1000 | 13,1 | 9,5 | -27,5 |

## Supplementary Figures


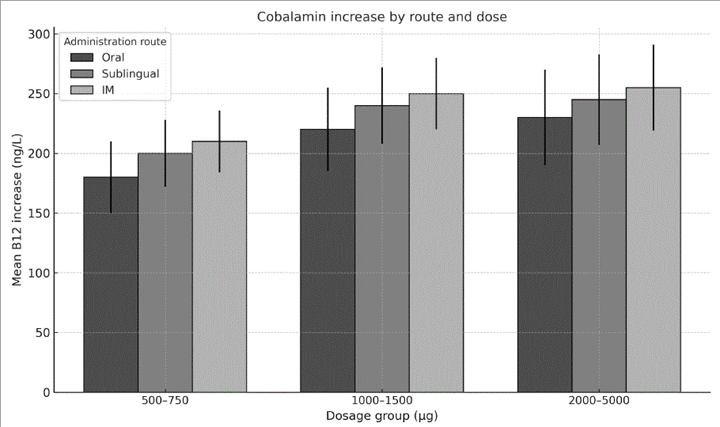


**Supplementary Figure S1.** Mean serum vitamin B12 (cobalamin) increase (ng/L) by administration route and dosage group. Comparison of oral, sublingual, and intramuscular (IM) vitamin B12 supplementation stratified by dosage range (500–750 µg, 1000–1500 µg, and 2000–5000 µg). Bars represent the mean increase in serum B12 levels with corresponding standard deviations.

**
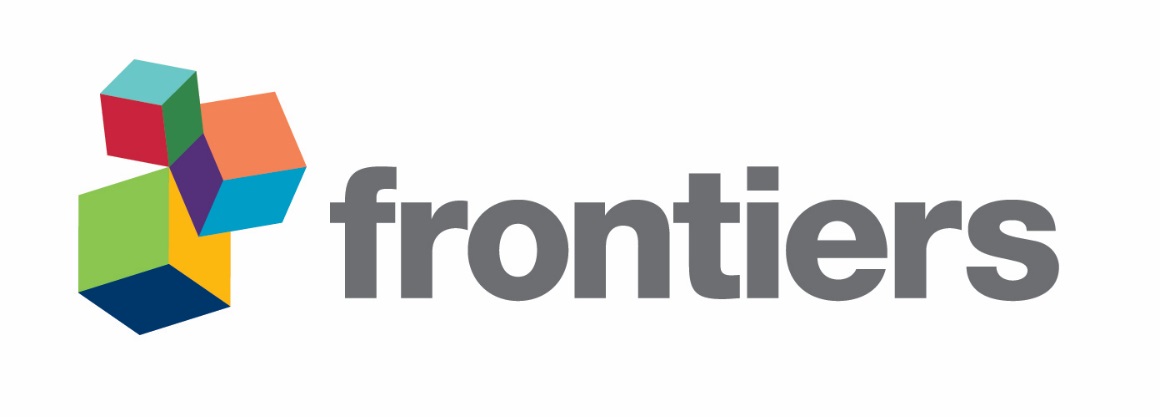
**
